# Hypoglycemia Induced by Insulin as a Triggering Factor of Cognitive Deficit in Diabetic Children

**DOI:** 10.1155/2014/616534

**Published:** 2014-03-23

**Authors:** Vanessa Rodrigues Vilela, Any de Castro Ruiz Marques, Christiano Rodrigues Schamber, Roberto Barbosa Bazotte

**Affiliations:** Department of Pharmacology and Therapeutics, State University of Maringá, 87020-900 Maringá, PR, Brazil

## Abstract

This paper provides an overview of insulin-induced hypoglycemia as a triggering factor of cognitive deficit in children with type 1 diabetes mellitus. For this purpose, databases from 1961 to 2013 were used with the objective of detecting the primary publications that address the impact of hypoglycemia on cognitive performance of diabetic children. The results obtained from experimental animals were excluded. The majority of studies demonstrated that the cognitive deficit in diabetic children involves multiple factors including duration, intensity, severity, and frequency of hypoglycemia episodes. Additionally, age at the onset of type 1 diabetes also influences the cognitive performance, considering that early inception of the disease is a predisposing factor for severe hypoglycemia. Furthermore, the results suggest that there is a strong correlation between brain damage caused by hypoglycemia and cognitive deterioration. Therefore, a more cautious follow-up and education are needed to impede and treat hypoglycemia in children with diabetes mellitus.

## 1. Introduction

The main objective in treatment of diabetes is to reach glycemic values which are the closest possible to those found in nondiabetic patients. The absence of a rigorous control of glycemia predisposes the patient to a series of chronic complications that include nephropathy, retinopathy, neuropathy, myocardial infarct, cerebrovascular accident, and peripheral vascular disease.

During insulin therapy, one of the most common adverse reactions is hypoglycemia. In general, during hypoglycemia there is an accentuated release of adrenaline from the adrenal glands, leading to autonomic symptoms: sudoresis, tremors, tachycardia, facial flush, and other symptoms. Besides the autonomic symptoms, there may also be symptoms linked to the lack of glucose in the brain (neuroglycopenia): vertigo, confusion, exhaustion, weakness, headaches, inappropriate behavior, which may be confused with drunkenness, lack of attention, vision abnormalities, convulsions similar to epilepsy, and coma [1].

The assessment and correction of hypoglycemia are indicated only for patients who present with Whipple's triad: signs and symptoms consistent with hypoglycemia, glycemia <70 mg/dL, and improvement or resolution of symptomatology after elevation of glycemia.

It is interesting to note that when hypoglycemia episodes are repeated in short periods of time, there is a loss of perception of the symptoms of hypoglycemia [2–5]. This phenomenon, also present in children [6, 7], which may be detected from the moment of the second episode of hypoglycemia [8, 9], has mechanisms which are not yet completely understood [10] and may disappear as adjustments in treatment to avoid the occurrence of new episodes of hypoglycemia [11, 12]. However, if there is no correction, there may be increased frequency, intensity, and duration of hypoglycemia considering that the return of perception depends on the appearance of a glycemic value lower than when the previous episode occurred [13]. Another important aspect related to loss of perception of hypoglycemia is the possibility that the patients with a better control of their glycemia (lower values for a prolonged time) have a greater tendency to suffer severe hypoglycemia [14].

When the hypoglycemia is intense (mental confusion, loss of consciousness, convulsions, and/or coma) and the patient needs the help of another person to recover from this condition, hypoglycemia is considered severe.

Severe hypoglycemia occurs very frequently in patients submitted to insulin therapy [15, 16]. It is interesting to observe that when the reports of severe hypoglycemia by the patient and close family members are compared, it can be noted that the patient detects later [17] and reports a lower number of severe hypoglycemia episodes [18].

Thus, considering that the brain uses glucose as a source of energy, hypoglycemia could cause alterations in brain activity [19, 20] and neuronal death in more severe cases, justifying the possibility of correlation between hypoglycemia and cognitive alterations [21].

The term cognition refers to mental processes (thought, memory, learning, intelligence, reasoning, attention, decision-making, visual perception, motor coordination, etc.) that the individual uses to acquire and manage information [22]. Therefore, we consider cognitive alterations to be the conditions in which one or more previously described mental processes come to be altered.

The cognitive alterations that can occur during hypoglycemia are not caused by the increased release of glucagon, adrenalin, noradrenalin, GH, and cortisol [23] nor even by excess insulin [24]. On the other hand, cognitive alterations may occur regardless of hypoglycemia, in situations of sleep deprivation [25], hypoglycemia induced by fasting [26], and hyperglycemia [27] and in children of mothers who presented with gestational diabetes [28].

It is also important to emphasize that there is no doubt as to the fact that cognitive alterations occur during insulin-induced hypoglycemia [29–47]. Cognitive alterations are detected even before the patient's perception of his/her condition of hypoglycemia [48, 49] and are heterogeneous even for patients submitted to insulin-induced hypoglycemia under controlled conditions [50–52]. Additionally, cognitive alterations suffer the influence of factors such as age [15–53], consumption of alcohol [54] or caffeine [55], alanine administration [56], prolonged fasting [57], presence of microvascular damage [58], and degree of hypoglycemia [59]. Another aspect to be considered is the cognitive function to be evaluated, since in the same individual there may be cognitive alterations in some tests and not in others [43, 60]. However, whether the occurrence of recurring episodes of insulin-induced hypoglycemia could cause permanent cognitive alterations is still unknown.

Therefore, based on the assumption that hypoglycemia would generate brain damage, it becomes necessary to know the factors related to hypoglycemia in triggering cognitive deficit. Nevertheless, the answer to this question is not simple since scientific literature pertaining to this theme is heterogeneous, particularly as to the different measuring methods for cognitive alterations. Therefore, it is necessary to analyze each case carefully, aiming at establishing the participation of factors related to hypoglycemia in the development of cognitive deficit, especially in children submitted to insulin therapy, since they are more susceptible to hypoglycemia.

## 2. Hypoglycemia as a Limiting Factor for Reaching Desirable Levels of Glycemia

Two important multicenter studies, the Diabetes Control and Complications Trial (DCCT) and the United Kingdom Prospective Diabetes Study (UKPDS), demonstrated the benefits gained with rigorous glycemic control in the treatment of diabetes. The DCCT [61] demonstrated that the maintenance of glycemia close to normal levels during intensive insulin therapy reduces the incidence and seriousness of chronic complications in type 1 diabetes. The UKPDS study [62] also demonstrated similar results in the treatment of type 2 diabetes.

However, in the DCCT trial [61], the patients presented with three times as many episodes of hypoglycemia relative to conventional therapy. In the UKPDS [62], the incidence of hypoglycemia in type 2 diabetic patients rose from 15 to 30% relative to conventional therapy. Therefore, the risk of hypoglycemia and its consequences represent the main limiting factor as to the effort in seeking an ideal glycemic control [63–66].

In general, the risk of hypoglycemia is greater when one compares insulin therapy with the insulin secretagogues (sulphonylureas, mitiglinides, diethyl-dipeptidase 4 inhibitors, or GLP-1 agonists) and therefore the risk of cognitive compromise will always be greater when insulin is compared to other antidiabetic agents.

## 3. Neonatal Hypoglycemia as a Possible Triggering Factor of Cerebral Damage and Cognitive Deficit 

The earlier the appearance of hypoglycemia episodes, the greater the possibility of brain damage and cognitive compromise. According to this observation, brain damage and cognitive deficit were associated with neonatal hypoglycemia: neonatal encephalopathy [67], lesion of the occipital lobe and cortex [68–70], and reduction of the circumference of the head [71]. According to these studies, cognitive deficit has been described in children with a history of neonatal hypoglycemia [72].

Neonatal hypoglycemia is caused by congenital hyperinsulinism that can extend hypoglycemic episodes beyond the neonatal period and is also strongly associated with brain damage and deficit in neurological development [73].

Therefore, there is greater predisposition towards brain damage at a structural and functional level in children who presented with early hypoglycemia since the episodes occur at a crucial moment in brain development. Additionally, we should consider that the younger the age, the greater the difficulty in verbally expressing symptoms, which makes them more vulnerable to the consequences of hypoglycemia [74].

It should be pointed out that the situations described in this section do not involve the administration of insulin and that neonatal diabetes is extremely rare, and its treatment, in general, does not involve insulin therapy [75, 76]. For these reasons, hypoglycemia associated with the administration of insulin and its potential as a trigger for brain damage and cognitive deficit in children will be covered more specifically in the next section of this review.

## 4. Insulin-Induced Hypoglycemia (IIH) as a Possible Triggering Factor of Cerebral Damage and Cognitive Deficit in Children

The first aspect to be pointed out is that the earlier the IIH episodes, the greater the risk of brain damage and cognitive deficit. According to this affirmation, it was noted that cognitive deficit is more evident in children with a history of severe IIH and who presented with the disease at less than 5 years of age [77–80].

On the other hand, risk of brain damage also extends to adults, since it was noted in these patients that hypoglycemic coma induced by insulin causes neuronal loss of white and grey matter of the telencephalon [81] and global brain atrophy [82]. Moreover, it is important to consider that, regardless of the occurrence of IIH, adult patients with type 1 diabetes in which the disease appeared at an age under 7 years presented with greater incidence of brain atrophy and intellectual performance relative to those in whom type 1 diabetes appeared at a later phase [83].

Another factor that contributes towards cognitive deficit is the greater frequency of severe IIH episodes [78–84], particularly when the patient presents with a history of convulsions [85]. In this aspect, it must be taken into consideration that cognitive alterations are more intense in children who present with a greater frequency of severe IIH [86].

Although the majority of studies cover separately the precociousness of type 1 diabetes appearance [83–87], the occurrence of episodes of severe IIH [87] and their frequency [78], and the intensity of the hypoglycemia [85], these four factors are strongly interconnected as the early appearance of type 1 diabetes leads to an earlier introduction of insulin therapy, increasing the possibility of the IIH episodes occurring more frequently and with greater intensity. Additionally, other factors that also increase the risk of IIH in children with type 1 diabetes should be considered, duration of type 1 diabetes, alterations in dose and type of insulin, insulin injection regime [88], and exercise [89], and belong to disadvantaged minorities [90].

## 5. IIH as a Possible Triggering Factor of Cerebral Damage and Cognitive Deficit in Children: An Issue Not yet Totally Clear

The first argument favorable to associating IIH with brain damage and cognitive deficit in children results from the fact that, in type 1 diabetic patients with a history of severe IIH, cognitive dysfunction is generally irreversible [91–97] and is caused by brain abnormalities [98–100]. For example, in adults, alterations in electric activity of the temporal lobe [101], cortical atrophy [102], and lesions of the left temporal lobe [103] were noted which would justify the lower performance of these patients in memory tests [78, 86, 104] and other evaluations of cognitive function [105].

Therefore, the fact that hypoglycemia causes brain damage and cognitive deficit in adults significantly reinforces the argument presented by studies in children with type 1 diabetes on insulin therapy, the majority of whom suggest the existence of cognitive compromise associated with IIH [106]. Nevertheless, some clinical studies [107–118] contrast with this position, since no cognitive deficit was observed in association with IIH in children. However, we cannot ignore the fact that, in one of these scientific articles [109], there was follow-up of 1144 type 1 diabetic patients over the course of 18 years and no evidence of cognitive function decline was found associated with a history of severe IIH episodes. It must be emphasized that although this paper published in The New England Journal of Medicine contrasts in terms of results with the majority of clinical studies relative to the theme, the number of patients evaluated (*n* = 1144) and the period of follow-up (18 years) exceed the other studies presented in this review. Nonetheless, it should be pointed out that this study included only type 1 diabetic patients on insulin therapy between 13 and 39 years of age, and, as was mentioned earlier, the greatest risks of hypoglycemia and cognitive deficit would be present in children who presented with the disease at less than 5 years of age [77–80].

Lastly, although it is not the focus of this review, another variable to be considered is the possibility of hypoglycemic episodes occurring within a context of hyperglycemia resulting from inappropriate insulin treatment, since it must be considered that acute [119, 120] and chronic [121–124] hyperglycemia, gestational hyperglycemia [125], and glycemic variability [126, 127] can also contribute towards the reduction of cognitive performance, since the excess of glucose can lead to brain damage regardless of the occurrence of IIH [128]. Moreover, studies in laboratory animals suggest that hyperglycemia after hypoglycemia, a condition frequent in poorly controlled diabetes, would lead to more neuronal death than isolated hypoglycemia [129].

## 6. Conclusion

Most clinical studies suggest that frequent and severe episodes of IIH would hinder learning in children [123, 130] and in some situations this is associated with dead-in-bed syndrome in type 1 diabetic patients [131]. Therefore, it is important to intensify monitoring [132] and education of the child with type 1 diabetes submitted to insulin therapy, seeking to reduce the risks of hypoglycemia, particularly nocturnal hypoglycemia [2], hypoglycemia associated with exercise [133], and IIH during pregnancy [134].

The fact that needs to be taken into consideration is that children have a greater risk of hypoglycemia relative to adults, presenting greater sensitivity to the dose of insulin administered and greater daily variability in terms of calorie ingestion and physical activity.

The association between the history of IIH and cognitive deficit involves multiple factors, especially intensity, frequency, and duration of IIH. Additionally, it was noted that the earlier the appearance of diabetes in children, the greater the tendency towards hypoglycemia episodes and consequently the greater the risk of deterioration of cognitive function.

Brain damage caused by hypoglycemia episodes is a factor of structural and functional nature that triggers cognitive deficit. Another relevant aspect is that among the brain areas affected the hippocampus would be among the most sensitive, which would explain the fact that memory is one of the most affected cognitive functions.

**Figure 1:**
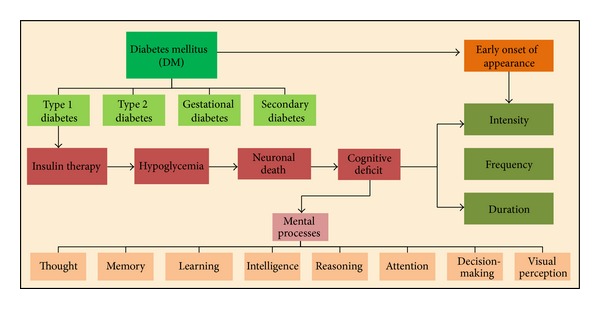
Insulin-induced hypoglycemia and its correlations with neuronal death and cognitive alterations.

A general view of the principal aspects covered in this review includes type 1 diabetes, hypoglycemia, neuronal death, and cognitive deficit, as well as the influence of factors such as precociousness in the appearance of type 1 diabetes, intensity, frequency, and duration of IIH, which are all summarized in Figure 1.
